# A Retrospective Study of Chemotherapy and 3D-Image-Guided Afterloading Intracavitary Radiotherapy in Locally Advanced Cervical Cancer

**DOI:** 10.1155/2022/9578436

**Published:** 2022-09-30

**Authors:** Xiaojun Li, Cunlian An, Chunlan Feng, Jieren Sun, Huixiang Lu, Xiaodong Yang, Kaiping Wang, Ruimei Wang

**Affiliations:** ^1^Heavy Ion Radiotherapy Department, Wuwei Cancer Hospital and Institute, Wuwei Academy of Medical Sciences, Gansu, China 733000; ^2^Department of Gynecology and Oncology, Wuwei Cancer Hospital, Gansu, China 733000

## Abstract

**Aim:**

To investigate the value of neoadjuvant chemotherapy combined with 3D-image-guided afterloading intracavitary radiotherapy in locally advanced cervical cancer (LACC).

**Methods:**

Patients with cervical cancer admitted to our hospital from January 1, 2020 to January 1, 2021 were retrieved and analyzed. Cases treated with neoadjuvant chemotherapy and 3D-image-guided afterloading intracavitary radiotherapy were assigned into the observation group (OG), while cases with neoadjuvant chemotherapy alone were assigned into the control group (CG). The short-term effects were determined by RECIST 1.1. Total effective rate (TR) = complete remission (CR) + partial remission (PR). The serum levels of squamous epithelial cell carcinoma antigen (SCC-Ag), glycoantigen 125 (CA125), carcinoembryonic antigen (CEA), and vascular endothelial growth factor (VEGF) were assessed. In view of the difference between tumor markers and diameters before and after treatment, the correlation between them was analyzed by Pearson test. The adverse events were compared, and the amount of operative bleeding and operation time were evaluated. Cox regression analysis was conducted to assess the influencing factors of 1-year disease-free survival time.

**Results:**

Sixty-seven patients were retrieved, including 30 cases in the OG and 37 cases in the CG. There were no significant differences in age, pathological type, tumor size, FIGO stage, past medical history, or smoking history between the two groups (*P* > 0.05). The TR of patients in the OG was higher than that in the CG (*P* < 0.05). The SCC-Ag, CA125, CEA, and VEGF levels in the OG decreased markedly after treatment (*P* < 0.001). The difference in SCC-Ag, CA125, CEA, and VEGF was positively correlated with the difference in tumor diameter before and after treatment (*P* < 0.05). The incidence of adverse events revealed no obvious difference between the OG and CG (*P* > 0.05). Cox regression analysis showed that FIGO stage and treatment regimens were independent prognostic factors for 1-year disease-free survival (*P* < 0.05).

**Conclusion:**

Neoadjuvant chemotherapy combined with 3D-image-guided afterloading intracavitary radiotherapy can improve the TR rate and 1-year disease-free survival of LACC patients without increasing the incidence of adverse events.

## 1. Introduction

Globally, cervical cancer (CC) ranks among malignancies with the highest number of new cases and deaths, posing a serious threat to the health of women [1]. In China, CC screening still needs to be popularized due to uneven regional healthcare development, and many patients are already in the stage of locally advanced cervical cancer (LACC) at initial diagnosis [2, 3], who are unable to be treated solely by surgeries [4, 5]. The 5-year survival rates of CC patients in stage IB1 and IIA1 are 80%-90% and 79.7%, respectively, while those of stage IB2 and IIA2 decreased to 50%-60% [6, 7].

Radical concurrent radiotherapy is the standard of care for advanced CC with NCCN guideline class 1 evidence, but the optimal treatment regimen for LACC is currently highly controversial, and there is no consensus worldwide [8]. The standard treatment recommended in the United States and Canada is concurrent radiotherapy, while countries in Europe, Asia, and Latin America use neoadjuvant chemotherapy followed by surgery as first-line treatment [9]. LACC patients are difficult to cure and have a poor prognosis due to the large localized extent of the tumor and the high risk factors [10]. For LACC with tumor diameter ≥ 4 cm, it is not easily controlled by surgical treatment alone and is prone to distant metastases and lymph node metastases after surgery [11]. Currently, the preoperative adjuvant treatment options mainly include neoadjuvant chemotherapy and radiotherapy. Radiotherapy is a local treatment, while chemotherapy can treat distant metastases and lymph node metastases while reducing the tumor [12]. Despite the international controversy regarding preoperative adjuvant therapy for LACC, preoperative adjuvant radiotherapy, neoadjuvant chemotherapy, or their combination are still popular in developing countries by reducing tumor volume, improving the tissue environment around the uterus, and facilitating surgical operation. Also, they can reduce the difficulty of surgery, improve the surgical resection rate of patients, and control tumors effectively [13]. Research has proven that combining radiotherapy with chemotherapy is an even more effective way to improve the local control rate of advanced CC [14]. However, radiotherapy alone can increase drug resistance and lead to many side effects.

To reduce the side effects of radiotherapy toxicity and to ensure efficient and sustainable treatment, it has been a hot topic of research in the gynecologic oncology field. In this study, neoadjuvant chemotherapy combined with 3D-image-guided afterloading intracavitary radiotherapy was offered to LACC patients prior to radical hysterectomy to observe the short-term clinical impact and outcome as well as adverse events, so as to evaluate the clinical significance of this regimen in future treatment.

## 2. Methods and Materials

CC patients treated at our hospital from January 1, 2020 to January 1, 2021 were analyzed retrospectively. Cases treated with neoadjuvant chemotherapy and 3D-image-guided afterloading intracavitary radiotherapy were assigned into the observation group (OG), while cases with neoadjuvant chemotherapy alone were enrolled into the control group (CG). All patients received radical CC surgery after treatment. The research was conducted with the approval of the medical ethics committee of Wuwei Cancer Hospital and Institute.

The inclusion criteria were cases confirmed through histopathology. The gynecologic examinations were performed by two gynecologic oncologists of associate chief physician or above and diagnosed according to the FIGO stage IB2 and IIA2 (FIGO staging 2009) [15]. Patients should not receive targeted treatment before this research. Patients' clinical data were complete. All of them were informed and signed an informed consent form. The exclusion criteria were as follows: cases with serious complications or underlying diseases that could not tolerate the treatment plan; cases complicated with other malignancies; surgical history of cervical disease; history of radiotherapy, chemotherapy, or antitumor therapy; infectious or metabolic diseases; abnormal blood clotting function; cognitive impairment or mental illness; patients with allergic symptoms of chemotherapeutic drugs; and patients during lactation or pregnancy.

### 2.1. Treatment Regimens

Patients received neoadjuvant chemotherapy with paclitaxel plus platinum, which was sensitive to CC. Specifically, paclitaxel 135-175 mg/m^2^ was given intravenously on day 1, and cisplatin 50-75 mg/m^2^ was given on days 1 to 3. The chemotherapy was administered at 3-week intervals for 2 cycles, during which symptomatic treatments such as hydration and antiemetic were routinely used. The 3D-afterloading intracavitary radiation therapy with 5.5-6 Gy each time was performed twice and completed within 1 week [16]. Radical hysterectomy and pelvic lymph node dissection was performed 2 weeks after adjuvant therapy.

### 2.2. Outcome Determinations

The main outcomes include: the near-term outcomes were compared by the Response evaluation criteria in solid tumors version 1.1 (RECIST 1.1) [17]. Total response rate (TR) = complete response (CR) + partial response (PR). The squamous cell carcinoma antigen (SCC-Ag), carbohydrate antigen (CA125), and carcinoembryonic antigen (CEA) of squamous cell carcinoma before and after treatment were tested by chemiluminescence method [18], and the level of serum vascular endothelial growth factor (VEGF) was determined by enzyme linked immunosorbent assay (ELISA). The correlation between tumor markers and diameter changes was assessed by Pearson's test according to the difference between patients before and after treatment.

The secondary outcomes include: the clinical characteristics and adverse events of both groups were compared. The amount of intraoperative bleeding and operation time were assessed. Cox regression analysis was conducted to assess the influencing factors of 1-year disease-free survival time.

### 2.3. Statistical Analysis

Data were analyzed through SPSS 20.0 (IBM Corp., Armonk, N.Y., USA). The enumeration data were expressed as *n* (%) and analyzed using the chi-squared test, and the measurement data were shown as mean ± standard deviation (SD) and evaluated by independent *t*-test. The association between tumor markers and diameter was assessed via Pearson test. Patients' disease-free survivals were plotted using the Kaplan-Meier survival curves, and then analyzed through log-rank test. The prognostic factor affecting patients' disease-free survival time was assessed through Cox regression. A two-tailed *p* value <0.05 indicated statistical difference.

## 3. Results

### 3.1. Comparison of Clinical Characteristics

**Figure 1:**
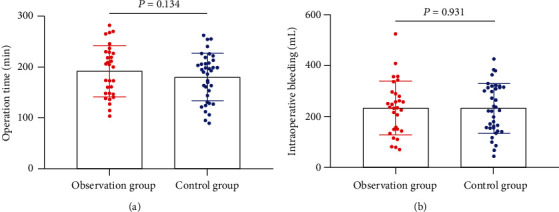
Blood loss and operation time during operation. (a) Difference of operation time between groups. (b) Difference of intraoperative blood loss between groups.

Sixty-seven CC patients were retrieved, including 30 cases in the OG and 37 cases in the CG. There were no statistical differences in age, pathological type, tumor size, FIGO stage, past medical history, or smoking history between the two groups (*P* > 0.05, Table 1). There was no marked difference in operation time and intraoperative blood loss between groups (*P* > 0.05, Figure 1).

### 3.2. Comparison of Near-Term Efficacy after Radiotherapy and Chemotherapy

There was significantly higher TR of patients in the OG than that in the CG (*P* < 0.05, Table 2).

### 3.3. Changes of Tumor Markers and VEGF Expression before and after Treatment

**Figure 2:**
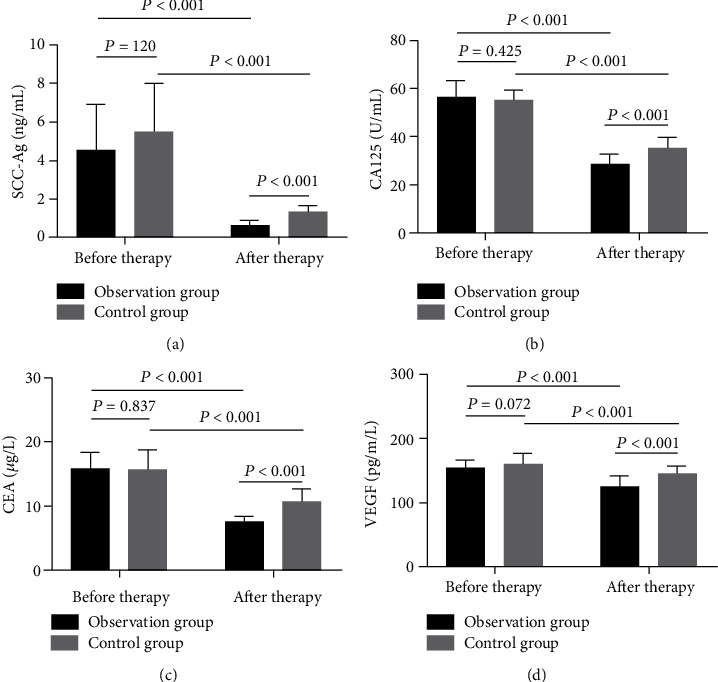
Comparison of serum tumor markers and VEGF levels in patients before and after treatment. (a) Comparison of serum SCC-Ag levels between groups before and after treatment. (b) Comparison of serum CA125 levels between groups before and after treatment. (c) Comparison of serum CEA levels between groups before and after treatment. (d) Changes of serum VEGF levels before and after treatment.

The changes of serum tumor markers and VEGF expression were compared after radiotherapy and chemotherapy before operation. The SCC-Ag, CA125, CEA, and VEGF levels in serum after treatment were lower than those before treatment (*P* < 0.001). After treatment, the levels of SCC-Ag, CA125, CEA, and VEGF in the OG were significantly lower than those in the CG (*P* < 0.001, Figure 2).

### 3.4. Correlation between Tumor Markers, VAGF, and Diameter

**Figure 3:**
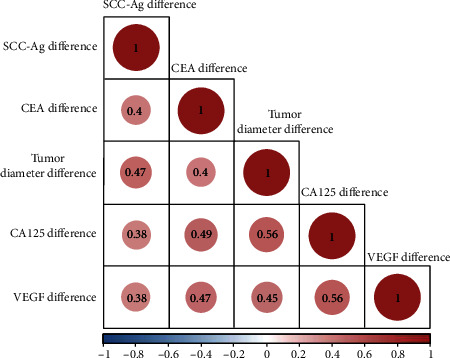
Correlation between tumor diameter, markers, and VEGF. Note: red indicates positive correlation, and blue indicates negative correlation.

We performed a correlation analysis based on the differences of indicators before and after treatment (Table 3). The differences of SCC-Ag, CA125, CEA, and VEGF before and after treatment were positively correlated with those of tumor diameter (*P* < 0.05, Figure 3).

### 3.5. Comparison of Adverse Events in Patients

There was no obvious difference in the incidence of adverse events between groups (*P* > 0.05, Table 4).

### 3.6. Analysis of Prognostic Factors of Disease-Free Survival Time

**Figure 4:**
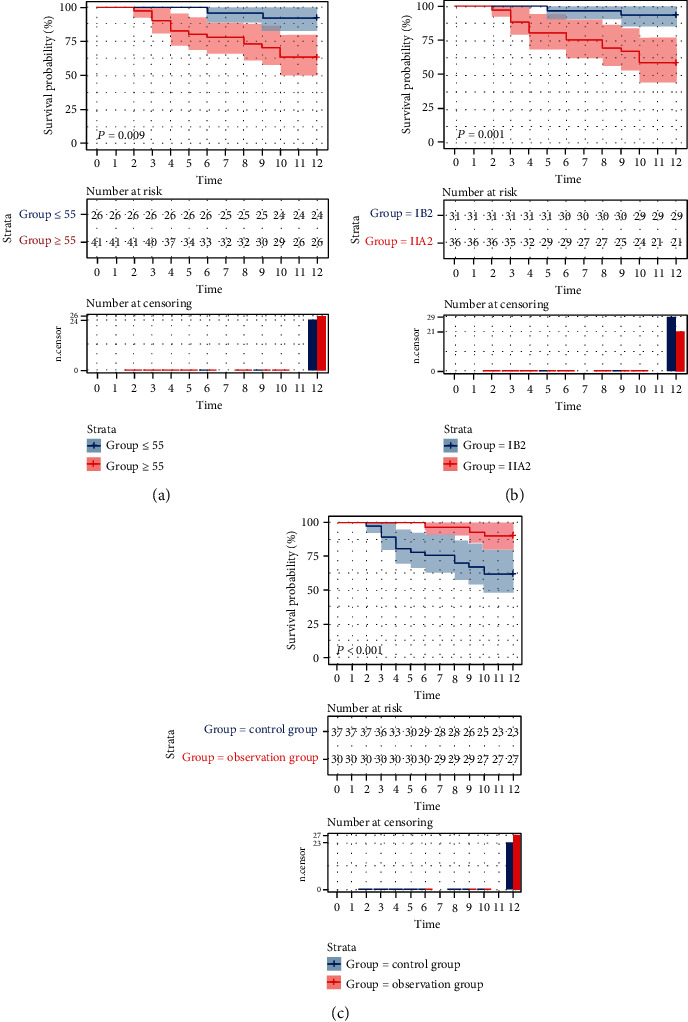
Analysis of age, FIGO stage, treatment plan, and disease-free survival of patients. (a) Analysis of age and disease-free survival of patients. (b) Analysis of FIGO staging and disease-free survival of patients. (c) Analysis of treatment plans and disease-free survival time of patients.

The 1-year disease-free survival rate of 67 patients was 74.62%. Subsequently, we analyzed the clinical data of patients using univariate analysis and found that age, FIGO stage, and treatment regimens were prognostic factors affecting disease-free survival (Figure 4). Further analysis revealed that FIGO staging and treatment regimens were independently tied to patients' disease-free survival (*P* < 0.05, Table 5).

## 4. Discussion

CC is a malignancy with high incidence in female patients. LACC accounts for a relatively large proportion among CC, and the 5-year survival rate is about 60% [19]. The tumor diameter of LACC patients is relatively large, which increases the difficulty of operation to a certain extent. The incidence of postoperative complications, metastasis, and recurrence rate are high, and the prognosis is not ideal [20]. Not only that, clinical treatment is difficult and more controversial. Although concurrent chemoradiation is considered an international standard treatment option, there are still many problems and limitations [21]. The number of patients treated tends to be younger, and radical surgery after preoperative adjuvant treatment is more in line with clinical needs. Neoadjuvant chemotherapy and 3D-image-guided afterloading intracavitary radiotherapy combines the advantages of precision radiotherapy and chemotherapy, so that CC with large tumor size can be well controlled, creating favorable conditions for surgical resection, and reducing surgical risks and complications. It improves the effect of treatment and quality of life of patients effectively [22]. Nevertheless, there are few studies on whether there is a difference in the efficacy between combination therapy and neoadjuvant chemotherapy alone in LACC treatment [23].

In the present study, we analyzed the efficacy of the two regimens in LACC patients. In our study, we found no marked effect of the two regimens on overall outcomes and adverse events. But our further analysis revealed that the TR rate of patients in the OG was higher than that of those in the CG. Previously, research found that 3D-image-guided afterloading intracavitary radiotherapy combined with chemotherapy improved the treatment outcome of advanced CC [24]. This is due to the fact that 3D conformal HDR brachytherapy can calculate the radiation dose received by the target area and surrounding normal organ tissues more accurately, which is conducive to the development of reasonable individualized treatment plans [25]. Neoadjuvant chemotherapy and radiotherapy have synergistic effects, acting on different cell cycles, respectively. Chemotherapy synchronizes cancer cells with radiotherapy-sensitive cycles, increases radiotherapy sensitivity while shrinking tumor cells, accelerates the apoptotic process of cancer cells, and reduces the chance of CC metastasis, thus, improving the histopathology [26]. Radiotherapy shrinks the local mass and leads to narrowing and occlusion of some capillaries and lymphatic vessels in the pelvis, which facilitates surgical operations and reduces the difficulty of surgery, thus, improving the efficiency of surgery [27]. We also found no difference in intraoperative bleeding and operative time during surgery. It is theoretically believed that combined treatment can reduce the local tumor volume and improve the parametrial tissue gap, which in turn reduces the difficulty of surgery. It showed that the combined treatment did not reduce the difficulty of the procedure, and we believe that the physiology of patients was diminished after the combined treatment. In addition, after the combined treatment, the reactive adhesion of lymphoid tissue and the para-uterine tissue fibrosis increased, thus, increasing the difficulty of the operation.

Currently, tumor markers such as SCC-Ag, CA125, CEA, and VEGF are of clinical value in CC diagnosis and treatment [28]. SCC-Ag is a relevant antigen reflecting the proliferation of squamous epithelial cells [29]. CA125 is one of the specific tumor markers, mostly found in adult pleura, endometrium, fallopian tube endothelium, and endocervical lining, and its expression is relevant to the tumor load in patients [30]. CEA may reflect the risk of tumor cell infiltration [18]. VEGF is an essential vascular endothelial growth factor for distant metastasis and tumor recurrence [31]. We found the SCC-Ag, CA125, CEA, and VEGF expression in LACC patients decreased after treatment. Besides, we confirmed a positive correlation between the difference of SCC-Ag, CA125, CEA, VEGF, and tumor diameter, which indicates that SCC-Ag, CA125, CEA, and VEGF are relevant to tumor growth. It is suggested that joint observation of changes in the levels of these markers may have vital monitoring value for assessing the disease progression and treatment efficacy.

At the end of the research, we measured patients' disease-free survival time. Cox regression analysis revealed that FIGO staging and treatment regimens were relevant to their disease-free survival. Earlier studies have shown that patients with higher FIGO stage have shorter disease-free survival time, which is consistent with our findings [32]. We first found that neoadjuvant chemotherapy combined with 3D-image-guided afterloading intracavitary radiotherapy was effective in improving the short-term disease-free survival of LACC patients. We believe this is due to the fact that preoperative radiotherapy shrinks the local mass. Moreover, preoperative radiotherapy can reduce local cervical tumors in varying degrees, which can not only eliminate tumor cells or reduce their activity, block tumor vessels, improve para-uterine infiltration, increase surgical resection rate but also reduce intraoperative dissemination and improve survival rate.

We found that neoadjuvant chemotherapy combined with 3D-image-guided afterloading intracavitary radiotherapy can increase the TR rate of LACC patients and improve their short-term disease-free survival. Nevertheless, there are still some limitations. First of all, the study period is relatively short. We were only able to count the disease-free survival time of patients for one year, and the effect of combined therapy on long-term overall survival and disease-free survival needs further study. Second, we only collected a relatively small number of patients for this study. Finally, this was a retrospective study, the results of which might be biased. We hope to continue to follow patients in subsequent studies and retrieve more patients to confirm our findings. It might be more intriguing to consider the combined therapy in other complicated cases, such as infection, hypoxia, fulminant hepatitis, or wound healing problems [33–39].

To sum up, neoadjuvant chemotherapy combined with 3D-image-guided afterloading intracavitary radiotherapy in LACC patients improves the TR rates and 1-year disease-free survival and does not increase the incidence of adverse events.

## Figures and Tables

**Table 1:** Clinical characteristics of enrolled patients.

Factor	Control group (*n* = 37)	Observation group (*n* = 30)	*P* value
Age			0.856
≥55 years old	23	18	
<55 years old	14	12	
Pathological type			0.554
Squamous cell carcinoma	33	28	
Adenocarcinoma	4	2	
Tumor size			0.293
≥ 5 cm	33	24	
< 5 cm	4	6	
FIGO stage			0.830
IB2	17	13	
IIA2	20	17	
Past medical history			
Hypertension	12	15	0.144
Diabetes	10	8	0.973
Smoking history			0.202
Yes	8	3	
No	29	27	

**Table 2:** Comparison of near-term efficacy [*n* (%)].

Groups	Complete remission	Partial remission	Stable	Ineffective	RR
Control group (*n* = 37)	4 (10.80)	10 (27.00)	21 (56.80)	2 (5.4)	14 (37.80)
Observation group (*n* = 30)	6 (20.00)	13 (43.30)	9 (30.00)	2 (6.70)	19 (63.30)
*χ* ^2^/*Z* value	-1.808				4.309
*P* value	0.071				0.037

**Table 3:** Difference of various indexes before and after treatment.

Indexes	Observation group (*n* = 37)			Control group (*n* = 30)		
	Before treatment	After treatment	Difference	Before treatment	After treatment	Difference
SCC-ag	4.54 ± 2.38	0.65 ± 0.25	3.95 ± 2.30	5.50 ± 2.55	1.36 ± 0.31	4.17 ± 2.53
CA125	56.59 ± 6.64	28.64 ± 4.21	27.95 ± 8.97	55.53 ± 4.06	35.47 ± 4.29	20.06 ± 6.39
CEA	15.85 ± 2.41	7.63 ± 0.89	8.23 ± 2.48	15.71 ± 3.08	10.76 ± 1.97	5.28 ± 2.79
VEGF	154.06 ± 11.65	125.15 ± 15.66	29.75 ± 18.66	160.36 ± 15.71	145.29 ± 11.76	20.95 ± 15.18
Tumor diameter	6.26 ± 1.20	4.51 ± 2.10	2.23 ± 1.66	6.22 ± 0.95	5.10 ± 1.67	1.51 ± 1.23

**Table 4:** Adverse events of patients.

Groups	Leukopenia	Malignant vomiting	Abnormal liver function	Fever	Total incidence rate
Control group (*n* = 37)	3 (8.10)	2 (5.40)	2 (5.40)	1 (2.70)	8 (21.62)
Observation group (*n* = 30)	2 (6.67)	2 (6.67)	2 (6.67)	0 (0.00)	6 (20.00)
*χ* ^2^ value					0.026
*P* value					0.871

**Table 5:** Analysis of risk factors for disease-free survival time.

Factor	Univariate analysis			Multivariate analysis		
	HR value	*P* value	95% CI	HR value	*P* value	95% CI
Age (≥55 VS <55)	5.617	0.022	1.284~ 24.578	4.346	0.053	0.983~ 19.208
Pathological type (adenocarcinoma VS squamous cell carcinoma)	0.951	0.947	0.217~ 4.160			
Tumor size (≥5 cm VS <5 cm)	0.815	0.747	0.234~ 2.836			
FIGO stage (IB2 VS IIA2)	0.127	0.006	0.029~ 0.558	0.144	0.011	0.033~ 0.637
Hypertension (yes VS no)	0.301	0.059	0.086~ 1.048			
Diabetes (yes VS no)	1.503	0.422	0.556~ 4.065			
Smoking history (yes VS no)	0.036	0.218	0.000~ 7.086			
Treatment plans (combination VS single)	0.218	0.017	0.062~ 0.759	0.188	0.009	0.054~ 0.657

## Data Availability

The data used to support the findings of this study are available from the corresponding author upon request.
